# Trastuzumab in combination with metronomic cyclophosphamide and methotrexate in patients with HER-2 positive metastatic breast cancer

**DOI:** 10.1186/1471-2407-6-225

**Published:** 2006-09-15

**Authors:** Laura Orlando, Anna Cardillo, Raffaella Ghisini, Andrea Rocca, Alessandra Balduzzi, Rosalba Torrisi, Giulia Peruzzotti, Aron Goldhirsch, Elisabetta Pietri, Marco Colleoni

**Affiliations:** 1 Unit of Research in Medical Senology, Department of Medicine, European Institute of Oncology, Via Ripamonti 435, 20141 Milan, Italy; 2 Division of Medical Oncology, Department of Medicine, European Institute of Oncology, Via Ripamonti 435, 20141 Milan, Italy

## Abstract

**Background:**

HER2/*neu *overexpression is linked to promotion of angiogenesis in breast cancer. We therefore tested the activity of the combination of Trastuzumab with metronomic, low dose chemotherapy with cyclophosphamide (CTX) and methotrexate (MTX) in metastatic breast cancer (MBC).

**Methods:**

Between April 2002 and June 2005, twenty-two patients with metastatic breast cancer with the presence of overexpression or amplification of HER2-/*neu*, all pre-treated with trastuzumab plus other cytotoxics, were treated with trastuzumab (6 mg/kg every three weeks) in combination with metronomic chemotherapy (MTX 2.5 mg, bid on Day 1 and Day 4 every week) and CTX (50 mg daily) (CM).

**Results:**

The 22 enrolled patients are evaluable: most had an ECOG performance status of 0 (17 pts), and all were pre-treated with chemotherapy for metastatic disease; 14 had progressive disease at study entry, and 11 had progressive disease during the last trastuzumab therapy. Metastatic sites included: lung (5 pts), liver (14 pts), bone (12 pts), lymph nodes (8 pts), central nervous system (CNS) (9 pts). We observed 4 partial remission (PR) (18%, 95% CI 5–40%), 10 stable disease (SD) (46%, 95% CI 24–68%), and 8 PD (36%, CI 17–59%). The clinical benefit (RP plus RC plus SD for ≥ 24 weeks) in all pts and in pts with disease resistant to previous trastuzumab therapy were 46% (95% CI, 24–68%) and 27% (95% CI, 6–61%), respectively. Median time to progression was 6 months and median duration of treatment was 5 months (range, 0,7 to 18.4 months and range, 1 to 18 months, respectively). Overall clinical toxicity was generally mild. Grade ≥2 reversible liver toxicity and leukopenia were reported in 5 and 3 pts, respectively.

**Conclusion:**

The combination of trastuzumab and metronomic chemotherapy is effective and minimally toxic in advanced breast cancer patients. The efficacy observed in patients with disease resistant to trastuzumab supports the need of larger trial to confirm a role of this combination to delay acquired trastuzumab resistance.

## Background

Malignant tumors secrete factors that enable them to trigger their own angiogenesis. The initiation of angiogenesis requires acquisition of the angiogenic phenotype through a series of molecular events leading to increased expression of angiogenic factors and down-regulation of natural inhibitors [1].

Her2/neu is a 185-kilodalton transmembrane receptor tyrosine kinase that belongs to the epidermal growth factor receptor family [2,3]. Tumor overexpression of HER2/neu is present in about 30% of patients with breast cancer and is associated with a worse histological grade, decreased overall survival and altered sensitivity to chemotherapeutic agents [4,5]. Recently, Her2/neu has been implicated in tumor angiogenesis. Experimental studies suggest that neutralizing antibodies against Her2/neu or EGFR results in down-regulation of angiogenesis, through VEGF gene suppression [6]. It is reported in the literature that such interaction occurs *via *abrogation of the increased synthesis of HIF1*a *(hypoxia inducible factor-1*a*) induced by c-erbB2 activation by ligands (i.r. heregulin) [7]. Moreover, a hypoxic-independent mechanims has been recently advocated in the angiogenetic involvement of HER2-/neu [8].

Trastuzumab (Herceptin^®^; Genentech, South San Francisco, CA), a recombinant humanized anti-erbB2/HER-2 monoclonal antibody (MoAb) used in erbB2-overexpressing breast carcinoma, has been shown to have antiangiogenic properties [9]. Trastuzumab can induce normalization and regression of the vasculature in an experimental human breast tumor which overexpresses HER2 in mice, by modulating the effects of different pro- and anti-angiogenic factors [9]. The combination of trastuzumab with chemotherapeutic agents (paclitaxel, docetaxel) has been shown to increase the efficacy of trastuzumab in reducing angiogenesis in erbB2-overexpressing cells more than either therapy alone both in animal models and clinical studies [10,11].

We previously demonstrated the antitumor activity of oral low-dose methotrexate and cyclophosphamide delivered as metronomic chemotherapy in metastatic breast cancer and we have shown the correlation with vascular endothelial growth factor levels [12,13]. Other authors previously showed that long-term, low dose chemotherapy could elicit an antiangiogenic effect [14].

Based on these considerations, we evaluated the activity and tolerability of the combination trastuzumab plus low-dose oral cyclophosphamide and methotrexate in patients with metastatic breast cancer with overexpression or amplification of Her2-/neu and pre-treated with trastuzumab.

## Methods

### Patient selection

Patients included were required to have histologically confirmed metastatic breast carcinoma that either had, or had not, progressed after a line of trastuzumab alone or in combination with chemotherapy for metastatic disease. Other inclusion criteria were: measurable disease, age ≤ 80 years, performance status ECOG < 3, adequate bone marrow reserve defined as white blood cells > 4,000 mm^3 ^and platelets > 100,000 mm^3^, adequate renal function (serum creatinine < 120 μmol/l) and hepatic function (serum bilirubin < 20 μmol/l, AST (SGOT) < 60 IU/l). It was mandatory that all patients had to have recovered from any prior chemotherapy, radiotherapy, or surgery before the start of treatment.

Each patient included in this study gave their written informed consent. This protocol was notified to Ethical Committee.

### Evaluation and treatment

Baseline evaluation included clinical examination, chest X-ray, liver ultrasound or CT scan, bone nuclear scan (plus segmental bone radiographs when bone scans were positive), ECG, echocardiography with LVEF evaluation, and complete biochemical and hematological tests. A complete blood count was repeated every 14 days and biochemical tests every 28 days. Cardiac assessment via echocardiography with LVEF evaluation was performed every 3 months.

HER2/neu overexpression was assessed on routinely processed, formalin-fixed, paraffin-embedded tissue by immunohistochemical investigations using an automated immunostainer (TechMate 500; Dako, Glostrup, Denmark) and a peroxidase-based detection system in kit form (ChemMate; Dako) according to the manufacturer's instructions. The primary specific monoclonal antibody used was clone CB11; Biogenex. The FISH test was performed on routinely processed, formalin-fixed, paraffin-embedded tissues using PathVision Her-2 DNA Probe Kit (Vysis Inc, Doweners Grove I11, and Inform HER-2 Gene Detection System, Ventana Medical Systems, Inc, Tucson, Arizona)

Patients were treated with MTX orally at a dose of 2.5 mg twice a day on day 1 and 4 every week (10 am, 5 pm) and CTX orally at a dose of 50 mg a day (9 am) in combination with Trastuzumab (Herceptin^® ^6 mg/kg every three weeks, after a loading dose of 8 mg/kg at the first administration)

### Side effects and response

Toxicity was evaluated according to National Cancer Institute's (NCI) Common Terminology Criteria for Adverse Events (CTCAE) version 2.0

Treatment was withheld and delayed for 1 week in cases of a neutrophil count of less than 1,000 mm^3 ^and/or platelet count less than 75,000 mm^3^. A 50% dose reduction in the total amount of drug administered in each cycle was prescribed after hematological recovery. In cases of a neutrophil count less than 1,500 mm^3 ^but > 1,000 mm^3 ^and/or platelet count less than 100,000 mm but >75,000 mm^3 ^therapy was administered with a 50% dose reduction in the total amount of drug administered in each cycle. Re-escalation of drug doses was attempted if close monitoring was possible.

In the event of grade ≥ 2 anorexia, nausea, vomiting, diarrhea, stomatitis, dryness of the mouth, epigastric pain, increase in transaminases, all therapy was postponed until symptoms subsided. A 50% reduction of CM was performed for the next cycle, with subsequent re-escalation to full dosage if tolerated. Any other non-hematological Grade 3 toxicity was managed by a 50% reduction of dosage in the next cycle, which was not commenced until full recovery had occurred. Cardiac function was monitored with echocardiography every 3 months and therapy with Herceptin was interrupted for values of Ejection Fraction (EF) below 50 or in cases of reduction of ≤ 10% from baseline value.

Assessment of response was conducted according to WHO criteria and was performed after every 8 weeks of therapy. CR was defined as the disappearance of all known lesions on two separate measurements at least 4 weeks apart. PR was defined as a reduction of each lesion by at least 50%. SD was defined as a decrease of less than 50% or an increase of less than 25% with no new lesions, and progressive disease as an increase of more than 25% or appearance of new lesions. Clinical benefit was defined as the proportion of patients who achieved CRs, PRs, or SDs for at least 24 weeks.

### Statistical analysis

The study was conducted with a two-stage design according to Gehan. In order to attain a power of 0.9 to detect a response rate of 20%, 11 patients are required in the first stage and if none demonstrates a response the study must be closed because of lack of activity. Because two patients responded, a further eleven patients were required to reach a precision of 0.1 in the estimate of the true response rate. Confidence intervals for the response rates were calculated using exact binomial methods.

Estimated curves of time to progression were plotted from the first day of treatment by the method of Kaplan and Meier; response duration was measured from the date of achievement of response. The association between categorical variables was assessed by the Fisher exact test.

## Results

Between April 2002 and June 2005, twenty two metastatic breast cancer patients were treated with the combination of trastuzumab and metronomic chemotherapy. All patients but one demonstrated overexpression of Her2-/neu (3+) (seven patients determined in the primary (32%) and fifteen patients in the metastatic sites (68%). The FISH test was performed in the tumor with overexpression of Her2/neu (2+) and resulted positive for amplification of the gene. Progressive disease at study entry was registered in 14 patients; among these, 11 patients had progressive disease during trastuzumab-containing regimens as the last treatment before study entry. Moreover, one patient had progressive disease during the last trastuzumab-containing therapy (not the last treatment before study entry) (Table 1). Previous trastuzumab-containing regimens are showed in Table 1. Median follow-up was 15 months. All patients had measurable disease.

Oral cyclophosphamide plus methotrexate in combination with trastuzumab produced 4 PRs providing an objective response rate of 18% (95% CI 5–40%). An additional 10 patients had stabilization of disease and of these, 6 patients showed long-term disease stabilization (SD after 24 weeks), providing an overall clinical benefit (CR + PR + SD after 24 weeks) of 46% (95% CI 24–68%). Previous treatments in patients achieving clinical benefit are summarized in Table 2.

Patients achieving clinical benefit were all pre-treated with ≥ 2 lines of chemotherapy for metastatic disease and with at least one line of Trastuzumab containing regimen. The clinical benefit in patients with progressive disease at study entry was 36% (95 % CI,13–65%) (Table 2). Two patients among those who achieved PR and three patients among those who achieved prolonged disease stabilization had progressive disease at study entry. In particular, 3 patients had progressive disease to trastuzumab-containing combinations and 2 to trastuzumab-non containing regimen. Moreover one patient had progressive disease to the last trastuzumab-containing therapy (not performed as the last therapy before study entry). The clinical benefit in patients who progressed during the trastuzumab-containing regimen (trastuzumab-resistant) was 27% (3 pts out of 11, 95% CI, 6–61%).

Exploratory analysis on features associated with the achievement of a clinical benefit showed a trend toward a larger percentage of clinical benefit among patients who received only one previous line of trastuzumab plus chemotherapy (62%, 8 out of 13) compared with those who received more than one previous line (22%, 2 out of 9)

Median time to response was 2 months and, among responding patients, the median duration of response was 8.8 (range: 1–12 + months.). Median time to progression was 6 months (range 0.7–18.4 +; 95% CI, 3.6 to inf) and median duration of treatment was 5 months (range, 1 to 18+ months).

A total of 149 months of therapy were administered, median per patient 5 months. Only 3 patients (13.6%) had delayed chemotherapy and only 3 (13.6%) reduced dosages of MTX. Reasons for delay and dose reduction was mainly leukopenia and increase in transaminases. Table 3 summarizes the side effects observed. Treatment was well tolerated. The most frequently encountered toxicity was grade II leukopenia, which was observed in 14% of the cases. Increase in transaminase values were registered in 8 cases (36%) (2 cases with grade III).

No cardiotoxicity was reported with the exception of one patient with decreasing value of ejection fraction (EF) to 51% (less than 10% from baseline value) after one month of the trastuzumab therapy plus CM and after more than 1 year of the other trastuzumab-chemotherapy combinations.

## Discussion

The combination of trastuzumab with chemotherapeutic agents is a well established approach for treatment of HER2 positive metastatic breast cancer. Preclinical models and subsequent clinical data have demonstrated an additive or synergistic effect of the combination with platinum salts, paclitaxel, docetaxel, vinorelbine or more than one drugs [15-18].

Unfortunately, the success of these combinations in responding patients is compromised by the development of acquired resistance [4]. The mechanism of resistance to trastuzumab in animal models is the consequence of heritable genetic alterations and involved different, independent mechanisms [19]. The opportunity, in patients with progressive disease, of continuing trastuzumab combined with a non cross resistant chemotherapeutic regimen is a crucial question regarding trastuzumab strategy. Data on restored efficacy of trastuzumab with further associations after failure are limited, although some activity was recently reported in patients with progressive disease during trastuzumab therapy regarding a further non cross resistant chemotherapeutic regimen in combination with trastuzumab [20-22].

Recently, Kerbel et al reported increased angiogenic activity, as demonstrated by the up-regulation of VEGF, in tumors resistant to trastuzumab therapy in murine models [19]. Using this model, the authors showed that eventual relapses during trastuzumab therapy can be significantly delayed by prolonged combination therapy with metronomic low-dose cyclophosphamide chemotherapy. Another important issue is the preliminary evidence for the superiority of combining metronomic chemotherapy (cyclophosphamide) versus standard maximum tolerated dose (MTD) chemotherapy in terms of efficacy and toxicity.

The current trial, the only clinical experience reported of metronomic chemotherapy plus trastuzumab for patients with advanced breast cancer, indicates a role for this combination to possibly delay acquired resistance to trastuzumab, and to treat patients whose tumor variants have acquired resistance. In our series all patients had received trastuzumab before commencing the combination of metronomic chemotherapy and trastuzumab and 14 patients had progressive disease at their inclusion in the study. The majority of them were progressive during trastuzumab-containing regimen (11 patients) as the last therapy before study entry. We observed a clinically significant activity in both patients with progressive disease at study entry and in patients who had progressive disease during the last course of trastuzumab containing therapy. We have considered whether these results might be due to the effect of metronomic chemotherapy alone or due to a synergistic effect of the combination. In previous studies in which metronomic chemotherapy was used alone, we observed an overall response of 10% and a global clinical benefit of 32% in pre-treated patients [12,13]. In the present study, we observed an overall response of 18% and a clinical benefit (RP plus RC plus SD for ≥ 24 weeks) of 46%, confirming a significant activity of the combination of metronomic chemotherapy plus trastuzumab. Treatment beyond progression represents a new paradigm in cancer therapy, although the indirect comparison of the two studies (not obtained in a randomized setting) and the nature of the present study cannot justify the extended use of trastuzumab after progression or the use of the combination of trastuzumab with metronomic chemotherapy instead of the single modalities alone. Moreover, eight patients started the study protocol after achieving clinical response to previous trastuzumab combination therapy. The possibility that trastuzumab alone could have allowed a clinical benefit similar to that obtained with trastuzumab plus metronomic chemotherapy is reasonable.

Additional studies to test the independent contribution of metronomic chemotherapy and trastuzumab when used after disease progression during trastuzumab are warranted.

Among patients with progressive disease at study entry we observed 2 objective responses and 3 cases of prolonged stabilization of disease indicating an activity in terms of clinical benefit in resistant patients (5 out of 14 patients, 36%) (Table 2). Moreover, in 6 patients we registered no evidence of progressive disease for at least 24 weeks indicating that a small but relevant fraction of heavily pre-treated MBC patients can achieve long term clinical benefit with this treatment option.

Among patients in whom progressive disease was recorded during the last course of trastuzumab containing therapy (11 pts) we observed 2 PR and 1 prolonged SD, for an overall clinical benefit of 27%. These results are particularly relevant since they support, in a clinical setting, the combination of metronomic low-dose cyclophosphamide and methotrexate plus trastuzumab, as a possible strategy for delaying or treating acquired resistance to trastuzumab in human breast cancer xenografts, as previously published [19], although the nature of our study and the small sample size do not allow definitive conclusions on this issue.

The majority of patients (12 patients, 54.5%) of our series were pre-treated with more than 2 lines of chemotherapy (in combination or not with trastuzumab) and among these, 5 patients had previously received 4 lines. The chance of obtaining a long term clinical benefit after more than 2 lines of chemotherapy in metastatic breast cancer is usually thought to be very poor, in particular for patients with HER2/neu positive MBC characterized by an aggressive disease course [23]. Moreover, in the present series, 9 patients had CNS involvement. Among these, 6 achieved clinical benefit. The inclusion of these patients, for whom a local treatment (radiotherapy in the majority of cases) was also planned or previously performed, was allowed due to the ability of both CTX and MTX to pass the BBB (blood-brain barrier) and to have activity in brain metastases from breast cancer [24], although there are not data of efficacy with the use of low dose metronomic chemotherapy.

The palliative goal of treatment in metastatic breast cancer and the achievement of symptomatic control and maintenance of quality of life are desirable treatment end points for the individual patient. Within this context the results achieved indicate that metronomic chemotherapy in combination with trastuzumab might represent a relevant additional therapeutic tool. In fact patients with HER2/neu positive MBC, where long term treatment with trastuzumab has been demonstrated to be feasible and safe [25], may benefit from combination with drugs characterized by a low incidence of toxic effects to allow the long term delivery of the combination.

As previously mentioned, in our series toxicity was mild. The profile of side effects was in keeping with previously published data on metronomic chemotherapy and included only 18% of grade >2 leucopenia or neutropenia, and only 27% of non hematologic toxicity. The addition of trastuzumab does not seem to increase the systemic toxicity and the individual tolerability. Only one patient, treated with trastuzumab for more than 1 year before the addition of CM, experienced a mild decreased of ventricular left ejection fraction (less than 10% from baseline value) and trastuzumab was interrupted.

## Conclusion

Low-dose, oral cyclophosphamide and methotrexate combined with trastuzumab demonstrated substantial efficacy in metastatic breast cancer and provided control of the disease (clinical benefit) in a significant proportion of patients. Although derived from a small non randomized study, our results could provide further support for the disputable approach of treating breast cancer patients with her2/*neu *positive disease using trastuzumab- containing therapies beyond progression. Due to the theoretical background and the promising results reported in this study, the value of the combination of metronomic chemotherapy and trastuzumab should be further explored in larger clinical trials focused on the debatable attitude to continue trastuzumab beyond progression.

## Abbreviations

CTX: cyclophosphamide

MTX: methotrexate

MBC: metastatic breast cancer

CM: cyclophosphamide + methotrexate

PR: partial remission

SD: stable disease

CNS: central nervous system

BBB (blood-brain barrier)

## Competing interests

The author(s) declare that they have no competing interests.

## Authors' contributions

LO conceived of the study, participated in design and coordination and prepare the manuscript, AC participated in the design of the study and in the data collection, RG participated in the data management, AR participated in the design of the study and performed the statistical analysis, AB participated in the data collection, RT participated in the data collection, GP participated in the data management, AG participated in the study design, EP participated in the data collection, MC conceived of the study, participated in design and coordination and prepare the manuscript

All authors read and approved the final manuscript.

Institutional funding was used by each authors for study design and conduction, data collection, statistical analysis, manuscript preparation

## Pre-publication history

The pre-publication history for this paper can be accessed here:



## Figures and Tables

**Table 1:** Patients characteristics

N. Patients	22
Median age, years (range)	48.9 (31–70)
Progressive disease at study entry	
No	8
Yes	14
Progressive disease to Her combination	7
Progressive disease to Her alone	4
Progressive disease to non Her containing therapy*	3
Number of sites	
1	1
2	9
≥ 3	12
Tumor sites	
Lung	5
Liver	14
Bone	12
Lymphnodes	8
CNS	9
Other	10
Previous treatment with Herceptin alone	1
Previous treatment with Herceptin in combination	16
1 line	8
2 lines	4
3 lines	4
Previous treatment with Herceptin alone and Herceptin in combination	5
2 lines	4
3 lines	1
Herceptin + Vinorelbine	11
Herceptin + Taxanes	15
Herceptin + Others	5
ER and PgR negative	15
ER and/or PgR positive	7
Ki 67	
<20%	1
≥20%	12
Unknown	9
Her2/Neu overexpression	
2+	1
3+	21

*one also progressed to the last trastuzumab-containing regimen

**Table 2:** Previous treatments in patients achieving clinical benefit

Best response	Progression at study entry	Previous lines of chemotherapy for metastatic disease	Previous Trastuzumab-lines for metastatic disease
SD ≥ 24 weeks	NO	3	3
SD ≥ 24 weeks	NO	4	1
SD ≥ 24 weeks	YES*	4	1
SD ≥ 24 weeks	NO	3	1
SD ≥ 24 weeks	YES	4	1
SD ≥ 24 weeks	YES**	4	2
PR	YES*	3	2
PR	NO	8	3
PR	NO	2	1
PR	YES*	5	2

* Progression at study entry to Trastuzumab-containing regimen

** Progression to the last Trastuzumab-containing regimen

**Table 3:** Side effects

	Grade 1	Grade 2	Grade 3
Leukopenia	1	3	-
Neutropenia	1	1	-
Nausea/Vomiting	2	-	-
Mucositis	1	-	-
Transaminases	4	2	2
Asthenia	2	1	-
Cardiac	0	0	0
